# Der Einsatz von Virtual-Reality Lernszenarien für Softskills-Trainings

**DOI:** 10.1365/s40702-021-00784-2

**Published:** 2021-09-15

**Authors:** Karin Zak, Stefan Oppl

**Affiliations:** Department für Weiterbildungsforschung und Bildungstechnologien, Donau-Universität Krems, Dr. Karl-Dorrek-Straße 30, 3500 Krems, Österreich

**Keywords:** Virtuelle Realität, Softskills-Trainings, Theorie der kognitiven Belastung, Kognitive Effekte, VR-Design, Virtual Reality, Softskills-trainings, Cognitive load theory, Cognitive effects, VR-design

## Abstract

Der Artikel zeigt den wirkungsvollen Einsatz von Virtual-Reality-Trainings im Softskills-Bereich besonders für Kommunikations‑, Präsentations- oder Verhandlungskompetenzen. Zunächst werden auf die Vorteile im Vergleich zum klassischen Setting eingegangen, wie etwa die Unabhängigkeit von einer physischen Präsenz von Trainingspartnern, was VR-Trainings in Zeiten der COVID-19 Krise noch attraktiver werden lassen. Nachdem auf das Risiko einer kognitiven Überlastung in einer VR-Umgebung eingegangen wird, wird ein Fallbeispiel eines VR-Szenarios im Softskills-Bereich, das in einem österreichischen Unternehmen eingesetzt wird, dargestellt. Die Ergebnisse einer quantitativen Erhebung zeigen, dass die Gestaltung der VR-Lernsequenzen mit einer niedrigen kognitiven Belastung und förderlich für den Lernprozess wahrgenommen wurde. Konkrete Faktoren, die zu diesem Ergebnis führten, wurden in einer anschließenden qualitativen Befragung erhoben. Der Artikel schließt mit Handlungsempfehlungen für den didaktischen Aufbau von VR-Szenarien, die aus den gewonnenen Erkenntnissen unter Einbeziehung einiger Gestaltungsprinzipien aus der Theorie der kognitiven Beanspruchung abgeleitet werden.

**Zusatzmaterial online:**

Zusätzliche Informationen sind in der Online-Version dieses Artikels (10.1365/s40702-021-00784-2) enthalten.

## Abstract (en)

The article shows the effective use of Virtual-Reality-scenarios to improve softskills especially for communication, presentation and negotiation. First, some advantages of VR-softskills-trainings compared to conventional settings are presented, especially the independence on physically available persons for training, which makes VR-trainings even more attractive in times of COVID-19. After discussing the risk of cognitive overload in VR-environments, a case study of a VR-softskills-training used in an Austrian company is presented. The results of a quantitative research show that the design of the VR-Scenario led to a perceived low cognitive load and was seen to be supportive for the learning process. Specific factors leading to that result were derived from a subsequent qualitative research. Finally, several recommendations for a possible didactic structure of VR-scenarios based on the research and considering some design principles from the theory of cognitive load theory are concluded.

## VR im Softskills-Bereich

Virtual-Reality-Trainings im Softskills-Bereich werden als innovativer Trainingszugang gesehen, deren wirkungsvoller Einsatz bereits in mehreren Studien gezeigt wurde (Mast et al. 2018). Zur Verbesserung von Kommunikationsfähigkeiten findet man Anwendungsbeispiele aus dem medizinischen Bereich, wie das Erlernen von klaren Anweisung zwischen OP-Mitarbeiter*innen (Cordar et al. 2017) oder die bewusste Wahrnehmung von non-verbaler Kommunikation zwischen Arzt/Ärztin und Patient*in (Kron et al. 2017). VR-Trainings werden auch zur Förderung der Alltagskommunikation von Personen mit Autismus (Politis et al. 2019) oder zur Auseinandersetzung mit interkultureller Kommunikation (Lane et al. 2013) eingesetzt. Innerbetrieblich finden sich effektive VR-Lernszenarien zum Training von Führungskräften, zur Simulation von Verhandlungsgesprächen (Broekens et al. 2012), zur virtuellen Vorbereitung auf Bewerbungsgespräche (Baur et al. 2013) oder zur Verbesserung von Präsentationsfähigkeiten (Mast et al. 2018). Aus den Rückmeldungen der Lernenden zeigt sich, dass die genannten VR-Simulationen geeignet sind, um sich auf verschiedene Gesprächssituationen vorzubereiten, Gesprächsverläufe zu reflektieren oder Erkenntnisse über die Wirkung von unbewussten Körperhaltungen zu erlangen.

VR-Lernszenarien im Softskills-Bereich werden einige Vorteile zugesprochen, allen voran können Lernende neue Verhaltensweisen mit virtuellen Trainingspartner*innen (simulierte Verhandlungspartner, virtuelle Job-Interviewer*in oder virtuelles Publikum) unabhängig von der Verfügbarkeit von realen Personen ausprobieren (Mast et al. 2018; Politis et al. 2019). Weitere Vorteile von VR-Softskill-Trainings im Vergleich zum klassischen Trainingssetting sind vor allem in sozialen Aspekten angesiedelt. Nicht nur, dass herkömmliche Rollenspiele häufig als nicht authentisch wahrgenommen werden (Lane et al. 2013), es wird auch das Verhalten der Lernenden durch die Anwesenheit von Zuhörer*innen oder durch richtungsbestimmende Anweisungen durch die Trainerperson stark beeinflusst. In sozialen Situationen verursacht die Tatsache, dass man von anderen Personen bewertet wird (etwa bei einem Job-Interview oder beim Halten von Präsentationen) häufig Stress (Bombari et al. 2015). In der VR-Welt können Lernende emotional herausfordernde Situationen in einer risikolosen Lernumgebung trainieren und ihre Trainingsbedingungen (etwa die Größe, Zusammensetzung und Reaktionen eines virtuellen Publikums) und verschiedene Stresslevels selbst wählen (Mast et al. 2018; Politis et al. 2019).

Der Einsatz von VR-Lernszenarien wurde durch die COVID-19 Pandemie zusätzlich gefördert (Singh et al. 2020), was auch die Herausforderungen in der Umsetzung stärker sichtbar machte (De Ponti et al. 2020). Bei der Gestaltung von VR-Lernszenarien besteht die Herausforderung vor allem darin, den eigentlichen Trainingsgegenstand in der Wahrnehmung der Teilnehmer*innen nicht durch Hürden in der inhaltlichen oder technischen Aufbereitung in den Hintergrund geraten zu lassen (idib.). Lernende müssen sich mit dem ungewohnten Setting auseinandersetzen, was die kognitive Belastung ansteigen lässt (Frederiksen et al. 2020) und so zu mangelhaftem Lernerfolg führen kann (Parong und Mayer 2018). Um die bildungsspezifischen Chancen und Möglichkeiten der VR-Technologie zu nutzen, ist eine bewusste Gestaltung des Lernarrangements erforderlich (Hellriegel und Čubela 2018), um der unerwünschten kognitiven Mehrbelastung entgegenzuwirken. Letztlich zeigt sich jedoch, dass es noch wenig etablierte didaktische Modelle für VR-Lernszenarien gibt (Zender et al. 2018).

Dieser Artikel adressiert diese Lücke aus der Perspektive der auftretenden kognitiven Belastung. Er diskutiert mögliche Auswirkungen von VR-Lernszenarien auf die kognitive Belastung der Lernenden und zeigt anhand einer Fallstudie aus der Praxis, welche Gestaltungsprinzipien für den didaktischen Aufbau von VR-Settings zur Vermeidung kognitiver Überlastung beitragen können. Die Ergebnisse bilden damit einen ersten Schritt hin zur expliziten Berücksichtigung von Effekten des Einsatzes von VR-Technologien im Design von Lernszenarien für Softskills-Trainings.

## Kognitive Beanspruchung in VR-Lernszenarien

Die technischen Entwicklungen der letzten Jahre und die damit einhergehenden leistungsfähigeren Geräte verleiten dazu, VR-Lernumgebungen als realitätsnahe und komplexe Lernerlebnisse zu gestalten, wodurch die Gefahr der kognitiven Überlastung steigt. In einer Studie von Frederiksen et al. (2020) wurden zum Training von chirurgischen Eingriffen durch angehende Ärzt*innen zwei Simulationsvarianten getestet. In einer immersiven VR-Variante (mit head-mounted Brille) sehen die Lernenden die gesamte Lernumgebung mittels 360° Video. Die Vergleichsgruppe sieht in einer desktop-basierten Simulation auf einem Bildschirm nur den Ausschnitt der zu operierenden Körperstelle.

Die Ergebnisse zeigen, dass die Lernenden in der immersiven Variante besonders in sog. Stressorphasen (Eintreten einer Blutung) einen deutlich höheren Anstieg der kognitiven Belastung (+43 %) erlebten, was sich auf eine Verdoppelung der Reaktionszeit auf eine sekundäre Aufgabe auswirkte. Zusätzlich zeigte die immersive Gruppe im Vergleich zur Desktop-Gruppe schlechtere Leistungsfaktoren (gemessen etwa an der zur Durchführung der Eingriffe benötigten Zeit – 533 statt 409 s – oder des simuliert aufgetretenen Blutverlustes – 190 statt 140 ml). Das wird damit erklärt, dass die immersive Lernumgebung mit zusätzlichen Ablenkungen (Instrumente und Nebengeräusche eines echten OP-Raumes, mehrfache Konversationen durch Personen) ausgestattet wurde, um eine möglichst reale Situation abzubilden. Die komplexe Elementinteraktivität führte jedoch zu einer höheren kognitiven Belastung, die vor allem in der initialen Lernphase hinderlich ist. Die Studie empfiehlt daher besonders für Lernanfänger*innen eine konventionelle (desktop-basiert) Simulation.

Eine Studie von Parong und Mayer (2018) zeigt ebenfalls, dass der Lerneffekt in einem immersiven VR-Szenario aufgrund zu vieler Details, die von den essenziellen Inhalten ablenken, schlechter ausfällt als in einer Desktop-Vergleichsgruppe (Parong und Mayer 2018). Allerdings zeigte eine im Anschluss an das Training durchgeführte Befragung, dass die VR-Gruppe eine höhere Motivation (Mittelwert von 5,93 statt 4,11 auf einer 7‑teiligen Skala) und gesteigertes Interesse (Mittelwert von 6,11 statt 3,32) erlebte. Durch Adaptierung des VR-Szenarios unter Berücksichtigung von Erkenntnissen aus der Theorie der kognitiven Belastung (u. a. Segmentierung in kleinere Lerninhalte und Integration von Reflexionsaufgaben in der realen Welt) konnte das Lernergebnis der VR-Gruppe ohne Einbußen beim Faktor Motivation gesteigert werden. Die Rolle der Aufbereitung der Lernaufgabe in VR-gestützten Lernumgebungen und insbesondere deren Reflexion und Verankerung in der realen Welt wird auch in (Makransky et al. 2021) betont.

Insgesamt wird abgeleitet, dass VR-Lernszenarien eine effektive Trainingsmethode sein können, wenn im Lern-Design die Erkenntnisse aus der Theorie der kognitiven Beanspruchung, die im Folgenden in ihren Grundzügen dargestellt sind, berücksichtigt werden.

## Theorien zur kognitiven Beanspruchung

Die in den 1980er-Jahren entwickelte Cognitive Load Theory (kurz: CLT) beschäftigt sich mit der begrenzten Kapazität des Arbeitsgedächtnisses von Lernenden (Sweller et al. 2019). Sie geht davon aus, dass Lernende nicht mehr als zwei bis vier Elemente gleichzeitig im Arbeitsgedächtnis bearbeiten können, ohne in eine kognitive Überlastung zu geraten (Van Merrienboer und Sweller 2010). Aus einer Weiterentwicklung der CLT entstanden die Cognitive Theory of Multimedia Learning (kurz: CTML), die sich auf das Ansprechen von mehreren ebenfalls beschränkten Wahrnehmungskanälen in multimedialen Lernszenarien fokussiert und die Cognitive-affective Theory of Learning with Media (kurz: CATLM), die sich mit dem Risiko einer kognitiven Überlastung in interaktiven, multimodalen Lernszenarien beschäftigt (Moreno und Mayer 2007).

Diese Theorien unterscheiden drei Arten von Belastungen des Arbeitsgedächtnisses: Unter „Extraneous Cognitive Load“ versteht man die Belastung durch lernirrelevante Elemente in nicht optimalen Lernmaterialien. Unter „Intrinsic Cognitive Load“ wird die kognitive Belastung durch die Komplexität des Lerngegenstandes (Anzahl der Elemente und deren Interaktivität) verstanden. „Germane Cognitive Load“ bezeichnet die kognitive Beanspruchung durch den eigentlichen Lernprozess.

Beim Lern-Design ist zusammengefasst darauf zu achten, dass die Lernenden einer geringen Belastung durch Extraneous Cognitive Load ausgesetzt sind, damit ausreichende Ressourcen für die Bewältigung des Intrinsic Cognitive Load vorhanden sind und der aktive Lernprozess, der den Germane Cognitive Load verursacht, in Gang gesetzt wird (Van Merrienboer und Sweller 2010).

## Fallstudie

Die kritische Rolle des Lern-Designs bei der Gestaltung von VR-Szenarien zur Vermeidung unerwünschter kognitiver Überlastung wird in der Literatur wie bereits beschrieben breit bestätigt. Während einzelne Arbeiten sich auch mit möglichen Einflussfaktoren des didaktischen Designs beschäftigen, etwa bei Mayer und Pilegard (2014) oder Buchner und Aretz (2020), bleibt die Auseinandersetzung damit Großteils auf konzeptueller Ebene oder wird nur unter Laborbedingungen untersucht. Dieser Artikel nähert sich der Herausforderung aus einer praktischen Perspektive an und untersucht die auftretende kognitive Belastung anhand einer realen Fallstudie. Die daraus abgeleiteten Erkenntnisse werden in der Folge den konzeptuellen Überlegungen aus der Literatur gegenübergestellt. Bei der vorliegenden Fallstudie handelt es sich um ein VR-Verkaufstraining für Mitarbeiter*innen eines österreichischen Unternehmens. Als Ziele für das VR-Training werden das Üben von alltäglichen Kundengesprächen, die Steigerung der Empathie und im Idealfall die Erhöhung der Quote für einen Verkaufsabschluss genannt. Die Konzeptionierung und technische Realisierung wurde von einem Beratungsunternehmen übernommen, während der fachliche Inhalt über Verkaufsberatung (z. B.: Fragetechniken und Umgang mit Einwänden) von einem externen Fachtrainer geliefert wurde. Zusätzlich wurden unternehmensinterne VR-Coaches ausgebildet, die die Mitarbeiter*innen beim VR-Training begleiten. Das VR-Lernszenario wird im Unternehmen seit 2019 eingesetzt. Mittels einer quantitativen Erhebung (*n* = 22, Erhebungszeitraum 08–09/2020) und einer zusätzlichen qualitativen Befragung (*n* = 3, Erhebungszeitraum 10/2020) wurde die erlebte kognitive Belastung der Anwender*innen und deren mögliche Einflussfaktoren untersucht, um Ansätze für VR-Designprinzipien abzuleiten.

### VR-Lernumgebung und technische Umsetzung

Beim Aufbau der virtuellen Lernumgebung wurde auf eine möglichst hohe Realitätsnähe geachtet, indem mehrere Sequenzen eines typischen Beratungsgespräches zwischen zwei realen Personen mittels 360°-Videos im Unternehmensumfeld aufgezeichnet wurden. Die Videos wurden danach mit Lernelementen und Aufgaben angereichert. Das VR-Szenario ist als immersive Variante ausgeführt (Betrachtung der virtuellen Szenen über eine Oculus Quest VR-Brille). Die Interaktionen werden mit einem Handsensor (im Sichtfeld erscheint eine virtuelle Hand) getätigt. Der Perspektiven- und Szenenwechsel wird durch Kopfbewegungen der Lernenden gesteuert. Der mögliche Bewegungsradius ist gekennzeichnet (wenn sich der Lernende außerhalb der Grenze bewegt, erscheint in der Brille ein markiertes Raster), damit sich die Lernenden während der immersiven Phasen sicher fühlen. Ein anwesender VR-Coach sieht auf einem Laptop, welche Szenen der/die Lernende gerade durchläuft und wählt manuell als „Wizard of Oz“ (Dahlbäck et al. 1993) die Reaktionen des virtuellen Gesprächspartners auf die getätigten Aussagen des Lernenden aus.

### Trainingsablauf und didaktisches Design

Das VR-Training findet im Einzelsetting gemeinsam mit einem realen VR-Coach statt. Die Lernenden tragen die VR-Brille nicht die volle Trainingszeit, sondern nehmen sie zwischen den einzelnen Szenen ab, um die Inhalte mit dem VR-Coach zu besprechen. Das gesamte Training dauert etwa eine Stunde, wobei die VR-Inhalte eine Länge von 15 min haben.

**Abb. 1:**
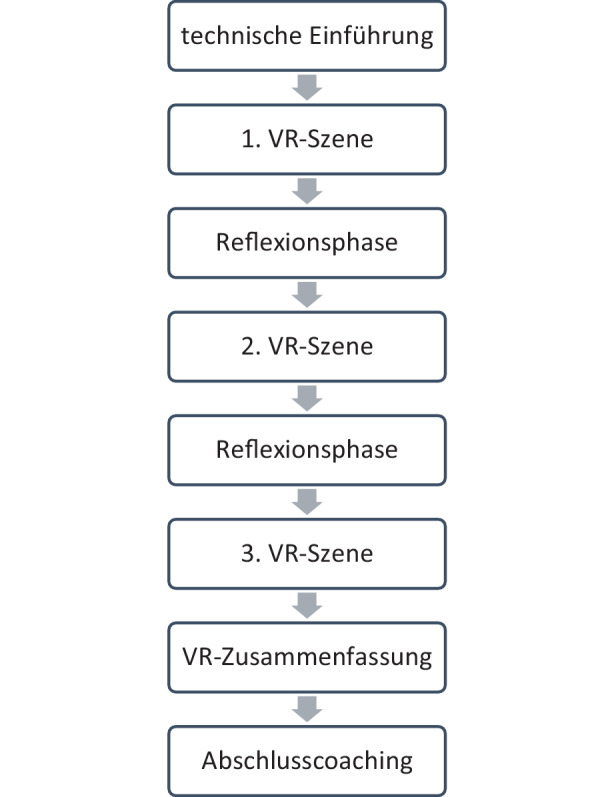
Ablauf des VR-Training

Das Training beginnt mit einer technischen Einführung (Setup, Start, Bedienung) durch den Coach und einer Eingewöhnungsphase. Der Inhalt ist in folgende 3 VR-Szenen geteilt (Abb. 1):

In der ersten VR-Szene betrachten die Lernenden zunächst ein Gespräch zwischen einem Berater und Kunde (Aufbau eines Gespräches und Bedarfserhebung). Die Beobachtung wird durch gezielte Fragen des Coaches („Welche Infos erhältst Du“, „Welchen Bedarf kannst Du beim Kunden feststellen“) gezielt geleitet. Die Lernenden bewerten die Qualität des beobachteten Kundengespräches mittels Handsensor. Zusätzlich erscheinen fachliche Begriffe im Blickfeld (Einblendungen), um die Aufmerksamkeit der Lernenden bewusst zu lenken. Danach kommt die erste Reflexionsphase, in der die VR-Brille abgenommen und mit dem Coach über den Inhalt und die getroffene Bewertung reflektiert wird. Zusätzlich werden fachliche Inputs (Produkte, Risiken, Begriffe) besprochen.

Die zweite VR-Szene (vertiefendes Beratungsgespräch) ist wieder mit einer Beobachtungs- und Bewertungsphase und einer anschließenden zweiten Reflexionsphase mit dem VR-Coach aufgebaut. In der dritten VR-Szene können die Lernenden selbst verschiedene Aussagen und Argumente ausprobieren, um auf Fragen und Einwände des virtuellen Kunden zu reagieren. Es gibt 3 manuell zu wählende Reaktionsmöglichkeiten des Kunden („Ich muss mir das noch überlegen“, „Jetzt haben Sie mich ganz verwirrt“, „Ich mache den Abschluss gleich“). Als Abschluss der VR-Szenen erscheint ein zusammenfassender Abschlusstext. Das Training wird mit einer Abschlussreflexion und einem individuellen Coaching (Festlegung der individuellen Stärken, Verbesserungspotential und Ziele) abgerundet.

### Empirische Untersuchung

Im Rahmen der Fallstudie wurde eine quantitative Erhebung mittels Online-Fragebogen durchgeführt, um die wahrgenommene kognitive Belastung der Teilnehmer*innen am VR-Training festzustellen. Anschließend wurde eine qualitative Befragung mittels leitfadengestützter Interviews durchgeführt, um die Ergebnisse aus der quantitativen Erhebung zu validieren und im Hinblick auf die kognitive Belastung konkrete störende bzw. förderliche Faktoren des VR-Designs zu erheben.

#### Quantitative Erhebung

Zur Messung der drei Arten der kognitiven Belastung, Intrinsic Cognitive Load (ICL), Extraneous Cognitive Load (ECL) und Germane Cognitive Load (GCL), wurde der von Klepsch et al. (2017) entwickelte Fragebogen mit 8 Aussagen für die vorliegende Studie in der Ausformulierung geringfügig adaptiert (siehe Tab. 1). Die Aussagen waren auf einer 7‑stufigen Likert-Skala (1 = „stimme überhaupt nicht zu“; 7 = „stimme voll und ganz zu“) zu beantworten.

**Tab. 1:** Fragebogen mit 8 Aussagen zur Erhebung der kognitiven Belastung

Item	Adaptierte Fragen für vorliegende Studie	Originalfragen lt. Klepsch (2017)
ICL1	Bei den VR-Lernsequenzen (mit Brille) musste ich viele Dinge gleichzeitig im Kopf bearbeiten	Bei der Aufgabe musste man viele Dinge gleichzeitig im Kopf bearbeiten
ICL2	Die Aufgaben in den VR-Lernsequenzen waren sehr komplex	Diese Aufgabe war sehr komplex
ECL1	Bei den VR-Lernsequenzen war es mühsam, die wichtigsten Informationen zu erkennen	Bei dieser Aufgabe ist es mühsam, die wichtigsten Informationen zu erkennen
ECL2	Die Darstellung in den VR-Lernsequenzen ist ungünstig, um wirklich etwas zu Thema Kundenberatung zu lernen	Die Darstellung bei dieser Aufgabe ist ungünstig, um wirklich etwas zu lernen
ECL3	Bei den VR-Lernsequenzen ist es schwer, die zentralen Inhalte miteinander in Verbindung zu bringen	Bei dieser Aufgabe ist es schwer, die zentralen Inhalte miteinander in Verbindung zu bringen
GCL1	Ich habe mich angestrengt, mir nicht nur einzelne Dinge zu merken, sondern auch den Gesamtzusammenhang zu verstehen	Ich habe mich angestrengt, mir nicht nur einzelne Dinge zu merken, sondern auch den Gesamtzusammenhang zu verstehen
GCL2	Die VR-Lernsequenzen enthielten Elemente, die mich unterstützten, das Thema Kundenberatung besser zu verstehen	Die Lerneinheit enthielt Elemente, die mich unterstützten, den Lernstoff besser zu verstehen
GCL3	Beim Bearbeiten der VR-Lernsequenzen ging es mir darum, alles richtig zu verstehen	Es ging mir beim Bearbeiten der Lerneinheit darum, alles richtig zu verstehen

**Abb. 2:**
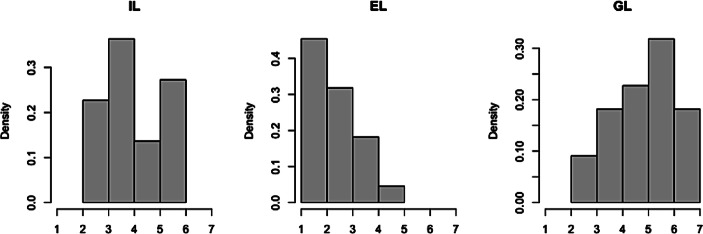
Histogramme der erhobenen Variablen; 7‑teilige Likert-Skala

Der Fragebogen wurde an 60 Mitarbeiter*innen, die das VR-Training innerhalb der letzten 18 Monate absolvierten, versandt (Erhebungszeitraum 08–09/2020). Alle vollständig ausgefüllten Fragebögen (*n* = 22, davon 11 männliche und 11 weibliche Teilnehmer*innen) wurden für die Analyse herangezogen. Aus den 22 Datensätzen wurde für jede Kategorie (ICL, ECL, GCL) und jedes Item der Mittelwert und die Standardabweichung errechnet sowie geprüft, ob die Ergebnisse signifikant vom Skalenmittelwert 4 (hypothetischer Median der 7‑teiligen Likert Skala) abweichen (Stichprobe genügt nicht den Anforderungen an einen parametrischen Test, deshalb Verwendung des zweiseitigen Wilcoxon-Tests, Signifikanzniveau *p* < 0,05). Tab. 2 zeigt eine Übersicht der Ergebnisse für die jeweilige Kategorie, Abb. 2 stellt die zugehörigen Histogramme dar.

**Tab. 2:** Ergebnisse der quantitativen Erhebung; 7‑teilige Likert-Skala, *n* = 22

Kategorie	Mittelwert	Standard abw	Signifikanz (*p* Wert)
ICL	4,14	1,13	0,501
ECL	2,39	1,06	0,000
GCL	4,94	1,33	0,007

Interpretation ICL/ECL: Je niedriger der Mittelwert desto niedriger die wahrgenommene Belastung

Interpretation GCL: Je höher der Mittelwert desto höher der kognitive Einsatz für den Lernprozess

Für die K**ategorie ICL** ergibt sich kein signifikant vom Skalenmittelwert abweichendes Ergebnis. Während das erste Item der Kategorie (ICL1) „*Bei den VR-Lernsequenzen (mit Brille) musste ich viele Dinge gleichzeitig im Kopf bearbeiten.“* eine signifikante Zustimmung aufweist, zeigt das zweite Item (ICL2) *„Die Aufgaben in den VR-Lernsequenzen waren sehr komplex.“, *dass für jeweils die Hälfte der Befragten die Aufgaben eher bis teilweise komplex bzw. eher bis überwiegend nicht komplex waren. Laut den Theorien zur kognitiven Beanspruchung hängt die wahrgenommene intrinsische Belastung maßgeblich von den Vorkenntnissen der jeweiligen Lernenden ab (Sweller et al. 2019), weshalb das Ergebnis möglicherweise auf heterogene Vorkenntnisse der Lernenden, die in der Befragung nicht systematisch erhoben wurden, zurückgeführt werden kann.

Für die **Kategorie ECL** und die einzelnen Items zeigt sich, dass die Befragten keine große Belastung durch Extraneous Load während des VR-Trainings wahrgenommen haben. Die VR-Einheiten waren so gestaltet, dass die Lernenden die wichtigsten Informationen leicht erkennen (Item ECL1) und die zentralen Inhalte in Verbindung setzen konnten (Item ECL3). Folglich sehen die meisten der Befragten das VR-Setting als geeignet, um etwas über das Thema Kundenberatung zu lernen (Item ECL2).

Für die **Kategorie GCL** gesamt und für zwei der drei inkludierten Items zeigen sich signifikant vom Skalenmittelwert abweichende Ergebnisse. Das VR-Training wurde als unterstützend für das Thema Kundenberatung wahrgenommen (Item GCL2). Die Befragten konnten ihre kognitiven Ressourcen so einsetzen, um sich beim Bearbeiten der VR-Lerneinheiten darauf zu konzentrieren, alles richtig zu verstehen (Item GCL3). Beim Item GCL1 *(„Ich habe mich angestrengt, mir nicht nur einzelne Dinge zu merken, sondern auch den Gesamtzusammenhang zu verstehen“)* konnte kein signifikantes Ergebnis gezeigt werden, was auf eine mögliche unterschiedliche Auslegung des Wortes „anstrengen“ als negatives Attribut und nicht als positive Bemühung zurückgeführt werden kann.

In einer offenen Frage haben 8 von 22 Befragten weitere Anmerkungen zum VR-Training angegeben. Dabei ist interessant, dass Personen mit explizit positiven verbalen Rückmeldungen in Bezug auf das VR-Training (*n* = 3) in der Kategorie GCL einen vom Gesamt-Mittelwert deutlich nach oben abweichenden Wert erreichen, d. h. diese Personen konnten mehr kognitive Ressourcen für den eigentlichen Lernprozess einsetzen. In den Kategorien ICL und ECL ist kein nennenswerter Unterschied zu verzeichnen. Personen mit explizit negativen verbalen Rückmeldungen (*n* = 2) zeigen eine höhere Belastung durch den unerwünschten Extraneous Load und konnten insgesamt weniger Ressourcen für den eigentlichen Lernprozess einsetzen, was sich durch eine geringere intrinsische Belastung zeigt. Eine mögliche Ursache für das Ergebnis könnte in der generell ablehnenden Einstellung gegenüber dem VR-Setting liegen, wodurch eine höhere Belastung in der als irrelevant bewerteten VR-Umgebung erlebt wird. Auch der Effekt des „Expertise Reversal“ aus der Theorie der kognitiven Belastung könnte hier zutreffen, d. h. die Lernaufgaben wurden als zu einfach erlebt, wodurch sich der Einsatz von kognitiven Ressourcen nicht lohnte (Leppink et al. 2013).

Zusammengefasst zeigt die quantitative Erhebung, dass das gewählte VR-Design im Hinblick auf die kognitive Belastung als gelungen bewertet werden kann. Wenngleich die Hälfte der Befragten eine eher hohe Belastung durch das Lernthema selbst erlebten (ICL), war die Aufbereitung der VR-Inhalte mit wenig lernirrelevanten Elementen ausgestattet (niedriger ECL), wodurch die Lernenden insgesamt ausreichend kognitive Ressourcen für den eigentlichen Lernprozess (GCL) einsetzen konnten.

In der anschließenden qualitativen Befragung, die im Folgenden dargestellt wird, wurden die für das Ergebnis ausschlaggebenden konkreten lernförderlichen bzw. lernhinderlichen Faktoren erhoben.

#### Qualitative Erhebung

Zur Triangulation und näheren Validierung der Ergebnisse aus der quantitativen Erhebung wurden Leitfadeninterviews mit Teilnehmer*innen des VR-Trainings geführt (Erhebungszeitraum 10/2020, 3 der Teilnehmer*innen der quantitativen Erhebung standen für ein Interview zur Verfügung). Die Interviews (Leitfaden mit 10 Hauptfragen) wurden über ein Online-Konferenztool geführt (Dauer von 17–36 min) und wurden im Anschluss transkribiert. Die Textstellen wurden mittels einer zusammenfassenden Inhaltsanalyse nach Mayring systematisch (Paraphrasierung, Generalisierung und Reduzierung) ausgewertet, um nachvollziehbare Ergebnisse hervorzubringen (Bortz und Döring 2006; Mayring 2019). Die reduzierten Aussagen wurden danach induktiv in 5 Hauptkategorien (VR-Umgebung, VR-Design, VR-Lernaktivitäten, VR-Mehrwert und VR-Weiterentwicklung) gebündelt.

Aus der Befragung ergab sich, dass die Orientierung in einer VR-Umgebung anfänglich gewöhnungsbedürftig ist. Zudem wird ein reibungsloser Szenenwechsel für eine gute Orientierung genannt (Probleme mit der Technik sowie stockende Szenenübergänge wurden als irritierend wahrgenommen).

Das gewählte VR-Design wurde als lernförderlich beurteilt, da der Gesamtablauf einer Kundenberatung in typische Szenen aufgeteilt wurde, die durch den hohen Praxisbezug für die Lernenden gut nachvollziehbar waren. Lernrelevante Grundbegriffe und Schlüsselwörter wurden im Blickfeld mit ausreichend Verarbeitungszeit eingeblendet, was für den Aufbau eines roten Gesprächsfadens als hilfreich erlebt wurde. Allerdings lässt sich aus der Befragung auch ableiten, dass fortgeschrittene Lerner*innen gewisse Einblendungen bereits als unnötig und irritierend empfinden können, wodurch zusätzlicher Extraneous Cognitive Load erzeugt wird, was in der Theorie als Expertise-Reversal-Effekt bekannt ist.

Die integrierten Szenen von Beobachtung und Bewertung von Musterdialogen wurden als förderlich zur Steigerung der Empathie (Hineinversetzen in die Kundensicht) genannt. Die Phasen der Selbstaktivität, bei dem der gezeigte Dialog von den Lernenden mit verschiedenen Reaktionsmöglichkeiten des Kunden fortgesetzt wurde, erlebten die Befragten als Variantenvielfalt, die man in einer realen Situation nicht vorfindet. Die Befragten gaben an, dass das VR-Szenario eine hohe Anzahl an Elementen, die im Kopf zu behalten sind (hoher ICL), enthält, die Verarbeitung der Elemente jedoch durch die ausreichenden Reflexionsphasen und den Austausch mit dem realen Coach unterstützt wurde.

Die Befragten nannten als besonderen Mehrwert des immersiven VR-Trainings (im Vergleich zu einer 2D-Desktop-Variante) die Möglichkeit des Eintauchens und Beobachtens einer virtuellen, aber realitätsnahen Gesprächssituation, wodurch das Gefühl der Präsenz gefördert wurde. Das VR-Szenario wurde als motivierendes Erlebnis wahrgenommen.

Bezüglich möglicher VR-Weiterentwicklungen wurden weitere Übungsvarianten zum vertiefenden Lerntransfer und Varianten mit zusätzlichen Stressoren hervorgehoben, was mit dem Effekt der „Variability“ zur Förderung des Lernfortschrittes aus der Theorie zur kognitiven Belastung im Einklang steht (Likourezos et al. 2019).

## Ableitbare Handlungsempfehlungen für den didaktischen Aufbau von VR-Lernszenarien

In diesem Abschnitt werden Handlungsempfehlungen für das didaktische Design von VR-Lernszenarien zusammengefasst, die sich aus der Fallstudie ableiten lassen und die in Verbindung mit den Prinzipien aus den Theorien zur kognitiven Belastung gebracht werden.

VR-Lernszenarien im Softskills-Bereich sind häufig in Phasen aufgebaut, die sich in (1) Auswahl und Darstellung der VR-Inhalte, (2) Zurechtfinden, Beobachten und Üben in der VR-Umgebung sowie (3) Feedback- und Reflexionsphasen einteilen lassen. Diesen Phasen werden im Folgenden abgeleitete Gestaltungsprinzipien zugeordnet.

### Auswahl und Darstellung der VR-Inhalte

Bei der Auswahl der Inhalte ist zunächst die Frage entscheidend, was mit dem VR-Setting erreicht werden soll. Das Lernziel eines Softskill-Trainings kann beispielsweise in der Verbesserung der Verhandlungsstärke von Lernenden liegen, wofür zuerst gewisse Verhandlungsregeln zu verstehen sind und danach verschiedene Verhaltensweisen ausprobiert werden sollen. Bei der Zusammenstellung der hierfür passenden Inhalte wird häufig auf die Fachexpertise von professionellen Trainer*innen zurückgegriffen (Broekens et al. 2012). Der komplexe Lernstoff kann danach im Sinne des Prinzips der Segmentierung in kleine zu erlebende Teilsequenzen aufgeteilt werden, wodurch die kognitive Belastung reduziert wird (Mayer und Pilegard 2014). Wie sich in der Fallstudie zeigt, müssen die einzelnen VR-Sequenzen einen nachvollziehbaren, roten Faden erkennen lassen (für Softskills-Trainings können die Szenen in typische Verhandlungsphasen oder Schritte zur Konfliktlösung geteilt werden). Die Nachvollziehbarkeit wird gefördert, wenn die VR-Situationen den Lernenden aus ihrer eigenen Praxis bekannt sind.

Generell ist bei der Inhaltsauswahl auf das Kohärenz-Prinzip (Sweller et al. 2019) zu achten, indem Elemente, die die VR-Lernumgebung zwar attraktiver erscheinen lassen, aber nicht lernrelevant sind, eliminiert werden. In dem Zusammenhang ist zu überlegen, wie realitätsgenau die VR-Umgebung sein muss, damit das Lernziel erreicht werden kann und sich die Lernenden noch mit ihr identifizieren können (Rau et al. 2019). In der Fallstudie wurde eine realitätsnahe Darstellung der VR-Szenen gewählt, wodurch laut der Befragten die Akzeptanz des Trainings gefördert wurde.

Bei der Darstellung der VR-Inhalte ist dem in den kognitiven Theorien gut erforschten Redundanzprinzip zu folgen, das heißt, eine gleiche Information soll nicht doppelt über verschiedene Darstellungsformen (etwa gesprochener und geschriebener Text) präsentiert werden (Moreno und Mayer 2007). Bei sehr komplexen Inhalten können jedoch wichtige Schlüsselwörter in Form von „Signaling“ eingeblendet werden, um die Aufmerksamkeit bewusst zu lenken (Moreno und Mayer 2010). In der Fallstudie wurden die Einblendungen von Schlüsselwörtern von den Befragten als richtungslenkende Denkanstöße wahrgenommen. Generell können in jeder VR-Phase Elemente zur Wissensvermittlung in Form von Einblendungen mit Zusatzerklärungen oder mittels einem On-Screen-Menü mit hinterlegten Ressourcen (beispielsweise generelle Kommunikationsregeln oder Informationen über die Wirkung von non-verbalem Verhalten) integriert werden (Broekens et al. 2012; Kron et al. 2017; Politis et al. 2019). In der Fallstudie folgte man dem aus der Theorie bekannten Kontiguitätsprinzip, d. h. alle Elemente wurden im räumlichen Blickfeld der Lernenden mit ausreichend Betrachtungszeit und zeitlich synchron mit einer Aufgabenstellung eingeblendet. Durch die räumliche und zeitliche Kontiguität müssen die Lernenden wenig kognitive Ressourcen zum Zusammensuchen der Informationsteile aufwenden, wodurch der Effekt der geteilten Aufmerksamkeit (Split-Attention-Effekt) vermieden wird (Sweller et al. 2019).

Wie im VR-Szenario der Fallstudie angewandt, kann durch den Einsatz von multisensorischen (auditive, visuelle und haptische) Elementen, die Überlastung eines einzelnen Wahrnehmungskanals im Sinne des Modalitätsprinzips verringert werden (Moreno und Mayer 2007; Rau et al. 2019; Sweller et al. 2019). Beim Einsatz von virtuellen Lernbegleitern, sog. pedagogical agents (Strohmann et al. 2018), die stilisiert sind, sollte auf menschliche non-verbale Gesten und Bewegungen geachtet werden (Rau et al. 2019). Zudem wirkt ein personalisierter Sprachstil, bei denen die Lernenden direkt angesprochen werden, motivierend und fördert die soziale Integration in die Lernumgebung (Mayer 2014).

Generell ist bei der Aufbereitung der Inhalte von VR-Szenarien auf einen reibungslosen technischen Ablauf sowie auf einen fließenden Übergang beim Szenenwechsel zu achten. Wie sich in der Befragung zur Fallstudie zeigt, erhöhen Probleme mit der Technik als irritierende Faktoren die kognitive Belastung. Bei Interaktionen mit virtuellen Gesprächspartnern sind, wenn eine automatische Spracherkennung nicht reibungslos funktioniert, daher technisch einfachere Lösungen (Auswahl über einen „Wizard of Oz“ oder ein Austausch über ein Auswahlmenü) zu bevorzugen (DeVault et al. 2015). Manuell geschaltete Interaktionen, wie sie in der Fallstudie verwendet wurden, wurden von den Befragten als wenig störend empfunden.

### Zurechtfinden, Beobachten und Üben in der VR-Umgebung

Wie sich aus der Fallstudie gezeigt hat, kann das Zurechtfinden in der virtuellen Umgebung anfänglich mit Unsicherheiten verbunden sein. Im Design sollte daher eine Eingewöhnungszeit berücksichtigt werden, in der die technische Handhabung mit der VR-Umgebung sowie Kopf- und Körperbewegungen ausprobiert werden können. Durch diese Vorabschulung wird die kognitive Belastung reduziert, was in der Theorie als Pre-Training-Effekt bekannt ist (Moreno und Mayer 2007).

VR-Lernszenarien im Softskills-Bereich beginnen oft mit Phasen des Beobachtens, in denen die Lernenden vorher erstellte Szenen betrachten und sich in die Rolle eines Mentors versetzen (Broekens et al. 2012; Lane et al. 2013). Im VR-Lernformat kann die schrittweise Demonstration von bestimmten Abläufen oder Musterdialogen (z. B. Best-Practice Verhandlungsdialog) gut umgesetzt werden, wodurch dem aus den kognitiven Theorien bekannten Worked-Examples-Effekt (Sweller et al. 2019) entsprochen wird. In der Fallstudie wurde das Einnehmen einer kritischen Beobachterrolle als lernförderlich angegeben. Die Lernenden konnten Rückschlüsse auf mögliche Verbesserungen im eigenen Verhalten ziehen, was zur Steigerung der Empathiefähigkeit beiträgt. In der Phase des Beobachtens ist zu berücksichtigen, dass ein rein selbstständiges Erforschen der Lernumgebung (discovery learning) eine hohe kognitive Belastung hervorrufen kann (Moreno und Mayer 2007). Die kognitiven Theorien empfehlen eine „Guided-Activity“, wodurch die Aufmerksamkeit der Lernenden gezielt angeleitet wird, was durch einen virtuellen Lernbegleiter oder durch On-Screen-Hinweise erfolgen kann (Rau et al. 2019). In der Fallstudie wurden die Lernenden zunächst über die Struktur des Trainings (besonders über den Wechsel zwischen VR- und realer Welt) informiert und während der Beobachtungsphase durch den VR-Coach gezielt mit Fragen angeleitet.

Nach den beobachtenden Sequenzen kann der Lernzuwachs durch Phasen von Selbstaktivitäten, wie etwa das selbstständige Weiterführen eines unterbrochenen Verhandlungsdialoges, gefördert werden. In der Fallstudie wurde das selbständige Fortführen eines Dialoges als besonders wertvoll betrachtet, was dem Problem-Completion-Effekt und dem Fading-Prinzip aus den kognitiven Theorien entspricht (Moreno und Mayer 2010).

VR-Lernumgebungen bieten die Möglichkeit, zu erlernende Fertigkeiten in verschiedenen Umfeldbedingungen (z. B. Präsentationen vor einem großen virtuellen Publikum mit störenden Elementen) und mit individuell wählbaren Parametern (z. B. zusätzliche Stressoren oder Variation der Realitätsabbildung) zu üben (Rau et al. 2019). Im Sinne der kognitiven Theorien fördern Übungsvarianten mit Schwierigkeitsgraden den Lernprozess, was als Variability-Effekt bezeichnet wird, wodurch auf das unterschiedliche Lernerniveau eingegangen werden kann (Sweller et al. 2019; Likourezos et al. 2019). Auch in der Fallstudie zeigt sich, dass die Lernenden die Variantenvielfalt an Situationen, die man in einem realen Trainingssetting nicht vorfinden kann, schätzen, weshalb diese beim Design von VR-Szenarien umgesetzt werden sollte. Um das selbstbestimmte Lernen zu fördern (Self-Management Prinzip), wird die Integration von Auswahlmöglichkeiten von individuellen Parametern empfohlen (Hellriegel und Čubela 2018; Sweller et al. 2019). In der VR-Umgebung können Optionen zum Navigieren, Suchen und Manipulieren (z. B. selbstgewähltes Lerntempo im Sinne vom Pacing-Prinzip oder Selbstansteuerung von einzelnen Szenen) eingesetzt werden (Parong und Mayer 2018).

### Feedback- und Reflexionsphasen

Entsprechend dem Feedback-Prinzip aus den kognitiven Theorien sollten den Lernenden Rückmeldungen auf getroffene Aktivitäten gegeben werden, um den Einsatz der kognitiven Ressourcen zu lenken (Moreno und Mayer 2010). In der VR-Umgebung kann implizites Feedback durch vorprogrammierte Reaktionen der virtuellen Partner (z. B. virtuelles Publikum zeigt Desinteresse, wenn der Lernende zu leise spricht) oder explizites Feedback durch Hinweise durch das VR-System (z. B. die Aufforderung, das Publikum häufiger anzuschauen oder die Gesprächsgeschwindigkeit zu ändern) integriert werden (Johnson und Priest 2014). Ein im VR-System integriertes Feedback kann allerdings die kognitive Belastung negativ beeinflussen, da der zusätzliche Input die Aufmerksamkeit beeinflusst (Mast et al. 2018). In der Fallstudie wurde das Feedback durch den VR-Coach gegeben und von den Befragten als wertvoll für den Lernprozess bezeichnet. In VR-Szenarien kann daher eine reale Trainerperson im Bereich des Feedbackgebens weiterhin eine essenzielle Rolle spielen.

Ein vertiefendes Lernen wird durch die Anreicherung der VR-Lernszenarien mit Reflexionsübungen unterstützt (Parong und Mayer 2018; Buchner und Aretz 2020; Makransky et al. 2021), wobei konkrete Anweisungen für die Lernenden hilfreich sind (z. B. wie der Aufbau der Gesprächsatmosphäre beurteilt wird). Reflexionsübungen können auch durch eine Verknüpfung der Phasen des Sehens in der virtuellen Welt mit Aktivitäten in der realen Welt umgesetzt werden (Hellriegel und Čubela 2018; Buchner und Aretz 2020). Für die Befragten in der Fallstudie trugen die eingesetzten Reflexionsübungen in der realen Welt unterstützend zur Bewältigung der vielen Elemente (Intrinsic Load) bei.

Schließlich sollte bei jeder Konzeptionierung von VR-Lernszenarien auch Möglichkeiten zur Messung der Effektivität geplant werden, was entweder durch eine Selbsteinschätzung der Lernenden (Broekens et al. 2012; Baur et al. 2013; Politis et al. 2019), die Vergabe von Score-Punkten für gewählte Optionen (Kron et al. 2017) oder durch die Analyse von bestimmten Faktoren wie beispielsweise die Wahl von Fragen und Antworten eines Lernenden, um einen Gesprächsfluss zu fördern (Broekens et al. 2012), realisiert werden kann. Zur Überprüfung des Lerntransfers sind Evaluierungstest nach einem gewissen Zeitverlauf nach dem Training denkbar (Kron et al. 2017). In der Fallstudie wurde eine Selbsteinschätzung der Lernenden als Rückmeldung auf das Training angewandt.

## Zusammenfassung und Ausblick

Insgesamt lässt sich aus der Fallstudie zeigen, dass die Einbettung des VR-Trainings in ein Gesamtarrangement mit Phasen der Beobachtung, der Selbstaktivität und der Reflexion als förderlich für den Lernprozess (Germane Load) wahrgenommen wird. Es kann gezeigt werden, dass die Ergebnisse aus den Erhebungen sich mit einer Reihe an Effekten und Prinzipien aus den Theorien zur kognitiven Belastung wie etwa „Worked-Example-Effekt“, „Fading“, „Reflection-Prinzip“ „Feedback-Prinzip“ „Guided-Activity-Prinzip“ und „Pre-training Effekt“ in Verbindung bringen lassen.

Zusammengefasst lässt sich schlussfolgern, dass das Potenzial der VR-Technologie nur sinnvoll genutzt werden kann, wenn gut durchdachte didaktische Konzepte, die über das reine explorative Lernen hinausgehen, eingesetzt werden (Hellriegel und Čubela 2018) und die Erkenntnisse der hier betrachteten Theorien zur kognitiven Belastung beachtet werden.

Die hier vorgestellten Ergebnisse sind unter der Limitation zu betrachten, dass die quantitative Erhebung auf einer relativ kleinen Anzahl an vollständig ausgefüllten Fragebögen basiert und die Homogenität der Befragten in Bezug auf ihre Erfahrungen angenommen aber nicht dezidiert erhoben wurde. Die Ergebnisse sind insofern als Hinweise auf mögliche Wirkzusammenhänge zu verstehen, die wissenschaftlich in repräsentativen Studien und unter der systematischen Erhebung weiterer Einflussfaktoren (z. B. Vorkenntnisse und Heterogenität der Teilnehmer, Motivation beider Gruppen etc.) bestätigt werden müssen.

Für zukünftige Studien zeigt sich aus der Fallstudie, dass es sinnvoll ist, Erhebungen über die kognitive Belastung unmittelbar nach jeder einzelnen VR-Szene durchzuführen. Weiters wären Effekte einer grundsätzlich ablehnenden Haltung gegenüber der VR-Technologie auf die kognitive Belastung im Vergleich mit Personen mit positiver Einstellung zu untersuchen, da sich in der Fallstudie hier bemerkenswerte Unterschiede zeigen. Die Forschung im Bereich von VR-Lernszenarien ist von hoher Dynamik geprägt, weshalb von vielen neuen Erkenntnissen in den nächsten Jahren auszugehen ist. Die COVID-19 Pandemie hat dem Einsatz von VR-Trainings einen zusätzlichen Entwicklungsschub gegeben. Die Nachhaltigkeit dieser Entwicklung für die betriebliche Praxis wird jedoch, wie im Artikel aufgezeigt, auch von der Umsetzung durchdachter didaktischer Konzepte abhängen, die die Prinzipien aus den Theorien zur kognitiven Belastung berücksichtigen.

## Supplementary Information

Anhang I: Interview-Leitfaden der qualitativen Erhebung
