# Supplementary material for: Using virtual-reality-based learning scenarios in soft skill trainings
Source: HMD Prax Wirtsch Inform. 2021 Sep 15;59(1):142–58. [Article in German] doi: 10.1365/s40702-021-00784-2 (PMC8442524; doi:10.1365/s40702-021-00784-2)
Supplement: Supplementary file 1 — Anhang I: Interview-Leitfaden der qualitativen Erhebung [file 40702_2021_784_MOESM1_ESM.pdf]

<u>Anhang I: Leitfaden für Interview</u>

Befragung von TN nach dem VR-Training

Einstieg:

- Wie lange sind Sie schon als Kundenberater tätig?

1. **Denken Sie sich noch einmal hinein: Wie das war, als sie die Brille aufhatten?**
    a. Wie haben Sie die **Orientierung** durch die VR-Einheiten (mit Brille) empfunden?
        - Wie waren die einzelnen Szenen für Sie aufgebaut (gut nachvollziehbar, *Segmentierung*)
        - Wie ist es ihnen beim **Szenenwechsel** (grauer, verschwommener Bildschirm) gegangen?
        - Es waren auch so **Schlüsselworte / Ergänzungen** eingeblendet. Waren die für Sie gut erkennbar? (visuelle Infos, und verbales Gespräch)?
        - Mussten Sie viele verschiedene Inputs **(Elemente) im Kopf** behalten?

2. **Wie gut konnten Sie sich in die Szene hineinversetzen?**
    a. Welche Elemente haben sie als eher störend oder irritierend empfunden (gab es etwas das eher zu Überlastung geführt hat, oder unnötig ablenkend)?
    b. Welche Elemente haben ihnen geholfen, mehr Sicherheit in einer guten Kundenberatung zu erwerben?

3. **Denken Sie noch einmal an die Aufgaben und Interaktionen, die sie gemacht haben.**
    a. **Es gab die Interaktionen mit dem Handsensor.** Welchen Eindruck nehmen sie hier mit?
    b. **Es gab den Teil, wo sie selbst Antworten gaben.** Wie haben Sie diesen Teil erlebt? (*Fading*) War da viel im Kopf zu behalten?
    c. Welchen Eindruck hat der **Wechsel zwischen der VR-Welt und den Fragen** vom echten Coach bei Ihnen hinterlassen?

4. **Was war bei den Musterdialogen für Sie besonders hilfreich?**
   a. Wie gut konnten Sie die Musterdialog und den Aufbau, die einzelnen Szenen nachvollziehen?
   b. Welche Dialoge (unterschiedliche Arten) hätten ihren Lernerfolg noch mehr gesteigert?

5. **Wie haben Sie die Anmerkungen vom e-Coach (Stimme aus dem off) empfunden?**
   a. Wie war es plötzlich eine Stimme zu hören? Konnten Sie diese Inputs, die sie gehört haben, gut verarbeiten?
   b. Waren die Anmerkungen hilfreich, motivierend, gaben sie Orientierung?
   c. Hätten Sie gerne den e-coach auch gesehen?

6. **Wie beurteilen Sie die Zeit, die sie hatten, um die verschiedenen Szenen zu beobachten?**
   a. Waren die eingeblendeten Informationen gut erkennbar, hatten Sie genug Zeit, diese zu verarbeiten?
   b. Wo hätten Sie sich noch mehr Selbstbestimmung gewünscht (Auswahl Szenen, Pausen, Tempo, Szenen wiederholen)?

7. **Stellen Sie sich vor, sie hätten den gleichen Inhalt am PC-Bildschirm als 2D Video** angesehen.
   a. Was wäre aus Ihrer Sicht der Unterschied gewesen (was wäre das für Sie anders gewesen)? *Immersion, Presence sollte der Unterschied sein*

8. **Was würden Sie verbessern/noch ergänzen, um beim Thema Kundenberatung noch tieferes Wissen zu bekommen?**

9. **Was müsste generell bei VR-Trainings noch besser sein?**

10. **In welchen Bereichen wünschen Sie sich noch den Einsatz von VR-Lernformaten?**
